# Adverse childhood experiences and academic burnout among Chinese traditional medicine students: the serial mediating role of rumination, self-control, and resilience

**DOI:** 10.3389/fpsyt.2026.1829698

**Published:** 2026-07-09

**Authors:** Youwen Wang, Shuailin Du, Ying Tan, Yuexuan Wu, Boying Yu, Xusheng Tian

**Affiliations:** 1 Graduate School, Heilongjiang University of Chinese Medicine, Harbin, China; 2 First Clinical Medical College, Heilongjiang University of Chinese Medicine, Harbin, China

**Keywords:** academic burnout, adverse childhood experiences, traditional Chinese medicine students, resilience, rumination, self-control

## Abstract

**Introduction:**

Medical students are vulnerable to academic burnout, particularly those with a history of adverse childhood experiences (ACEs). Although early adversity has been linked to burnout, the precise psychological processes underlying this association remain insufficiently understood. This study examined whether rumination, self-control, and resilience were statistically associated with the relationship between ACEs and academic burnout among medical students.

**Methods:**

A cross-sectional questionnaire survey was conducted among 1,889 medical students from Heilongjiang University of Chinese Medicine. Measures included ACEs, academic burnout, rumination, self-control, and resilience. Pearson correlation analysis, and serial mediation analyses were performed. Sensitivity analyses were conducted using alternative ACE codings, and supplementary analyses were used to compare alternative mediator orderings.

**Results:**

ACEs were positively associated with academic burnout (β = 0.187, p < 0.001). Rumination, self-control, and resilience were each statistically linked to the ACEs-burnout association through significant indirect pathways. After inclusion of the mediators, the direct association between ACEs and academic burnout was no longer significant. The overall pattern was broadly consistent across continuous, binary, and ordinal ACE operationalizations. Supplementary analyses further indicated that several alternative mediator orderings were also statistically plausible.

**Conclusion:**

These findings suggest that rumination, self-control, and resilience are statistically associated with the relationship between ACEs and academic burnout. However, because the study was cross-sectional, causal ordering cannot be established. Longitudinal studies are needed to clarify the temporal sequence of these psychological processes and to determine whether targeting them may help reduce academic burnout.

## 1 Introduction

Academic burnout has become an important mental health concern in medical education. Medical students are required to cope with intensive coursework, prolonged training, frequent assessment, and early exposure to clinical responsibilities (1). In the present study, academic burnout was conceptualized according to the Oldenburg Burnout Inventory Student version, which assesses exhaustion and academic alienation or disengagement (2, 3). Exhaustion refers to persistent physical, emotional, and cognitive fatigue related to academic demands (4). Academic alienation or disengagement reflects psychological distancing from learning, reduced identification with academic work, and negative attitudes toward study (2). This conceptualization is particularly relevant to medical and traditional Chinese medicine students, whose training environments require sustained cognitive effort, self-regulation, and professional commitment (5). Recent systematic reviews and empirical studies have shown that burnout is common among medical students and is associated with poorer mental health, impaired academic functioning, and unfavorable professional development (1, 6, 7).

Adverse childhood experiences (ACEs) may represent an important distal risk factor for academic burnout. ACEs refer to potentially traumatic or stressful experiences occurring in the early family environment, including childhood abuse, neglect, and household dysfunction (8, 9). In this study, ACEs were treated as a cumulative index of early-life risk exposure rather than as a single psychological trait (10). From a developmental perspective, repeated exposure to early adversity may affect later emotion regulation, cognitive processing, interpersonal functioning, and adaptation to academic stress (11). Studies in health science and medical student populations have also suggested that ACEs are associated with psychological vulnerability and health-related behaviors during medical training (12, 13). Although ACEs have been linked to a broad range of mental health and behavioral outcomes, the psychological mechanisms through which early-life adversity is associated with current academic burnout among medical students remain insufficiently understood.

Rumination, self-control, and resilience may help explain the association between ACEs and academic burnout. Rumination refers to repetitive and passive attention to distress, its possible causes, and its consequences (14). It may include symptom rumination, brooding, and reflective pondering (15). In academic contexts, elevated rumination may prolong negative affect and interfere with flexible problem solving, thereby increasing vulnerability to academic exhaustion and disengagement (16). Self-control refers to the capacity to regulate impulses, emotions, attention, and behavior in the service of longer-term goals (17). In the present study, self-control included self-discipline and impulse control, which are closely related to maintaining study routines, resisting distractions, and persisting with demanding academic tasks (18, 19). Resilience reflects a broader adaptive capacity to recover from stress and maintain functioning under adversity (20). In Chinese populations, resilience has commonly been described through tenacity, strength, and optimism (21). Recent studies have shown that resilience is negatively associated with student burnout, whereas maladaptive cognitive and emotional processes, including rumination, are associated with higher academic burnout (5, 16, 22, 23).

The Conservation of Resources theory provides a useful framework for integrating these variables. This theory proposes that individuals strive to obtain, maintain, and protect valued resources, and that stress occurs when resources are threatened, lost, or insufficiently replenished (24). From this perspective, ACEs may be understood as distal experiences of resource loss or resource deprivation (10, 25). Rumination may represent a resource-consuming cognitive process, whereas self-control and resilience may reflect different levels of current psychological resources (17, 20, 24). We therefore specified a theoretically informed serial mediation model in which rumination, self-control, and resilience were examined as statistically linked mediators in the association between ACEs and academic burnout. The ordering of these mediators was based on theoretical and developmental considerations. ACEs are early-life exposures that precede current psychological functioning. Rumination represents a relatively proximal negative cognitive-processing pattern (14). Self-control reflects current executive regulatory resources (17). Resilience reflects a broader adaptive resource that may be related to students’ ability to maintain functioning under stress (20, 26). However, because the present study used a cross-sectional design, this ordering should not be interpreted as definitive evidence of temporal or causal sequence. To examine whether other pathways were also plausible, we compared the hypothesized model with theoretically reasonable alternative sequence models in supplementary analyses.

Accordingly, this study aimed to examine the association between ACEs and academic burnout among Chinese medical students and to investigate the mediating roles of rumination, self-control, and resilience. We hypothesized that ACEs would be positively associated with academic burnout. These hypotheses were evaluated as statistical associations rather than causal pathways.

## 2 Materials and methods

### 2.1 Study design

**Figure 1:**
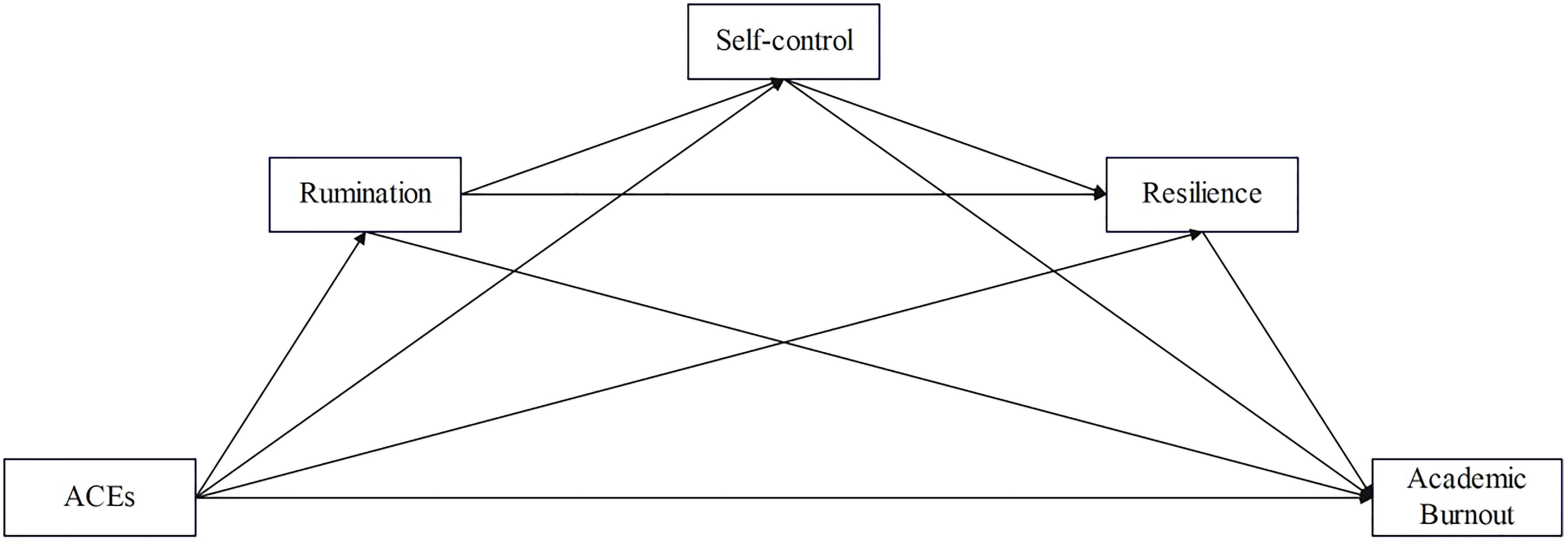
Hypothesized serial mediation model of the associations among adverse childhood experiences (ACEs), rumination, self-control, resilience, and academic burnout. This figure illustrates the proposed associations among adverse childhood experiences (ACEs), rumination, self-control, resilience, and academic burnout among medical students. The model was specified *a priori* based on resource conservation theory and examined using cross-sectional data.

This cross-sectional study was conducted to examine the association between ACEs and academic burnout, as well as the statistically linked roles of rumination, self-control, and resilience. The hypothesized serial mediation model is shown in **Figure 1**. Because all variables were measured at the same time point, the hypothesized ordering of the variables was theory-informed and was not interpreted as evidence of temporal or causal sequence.

### 2.2 Study participants and data collection

Convenience sampling was used to recruit clinical medical students from different academic years at the First Clinical Medical College of Heilongjiang University of Chinese Medicine. Data were collected anonymously using the *Wenjuanxing* online survey platform between September 16, 2025, and October 16, 2025. The survey was disseminated through university administrative channels and class-specific WeChat groups.

This study was approved by the Ethics Committee of the School of Humanities and Management at Heilongjiang University of Chinese Medicine (Approval No.: HLJZYYDXRWYGLXY20250105). Before starting the questionnaire, all participants were required to read an electronic informed consent form. Participation was voluntary, and completion of the questionnaire was considered to indicate informed consent. All responses were anonymous, and confidentiality was strictly maintained throughout the study.

### 2.3 Measures

#### 2.3.1 Demographic variables

The demographic questionnaire collected four variables, sex, residence, only-child status, and grade.

#### 2.3.2 Adverse childhood experiences (ACEs)

ACEs were assessed using the 10-item CDC-Kaiser ACE Scale, which was originally developed to assess major categories of childhood abuse, neglect, and household dysfunction (8). Abuse included emotional, physical, and sexual abuse. Neglect included emotional and physical neglect. Household dysfunction included parental separation or divorce, domestic violence, household substance abuse, household mental illness, and incarcerated household members.

Each ACE item was coded as 0 = no and 1 = yes. The 10 items were summed to generate a cumulative ACE count ranging from 0 to 10, with higher scores indicating exposure to a greater number of childhood adversities. This cumulative ACE count was used as the primary independent variable in the main regression and mediation analyses. Cronbach’s α was not calculated for the ACE questionnaire because ACEs are commonly conceptualized as a cumulative risk index of heterogeneous adverse exposures rather than as a unidimensional latent psychological construct (9, 11). To evaluate the robustness of the findings, sensitivity analyses were conducted using binary ACE coding, defined as 0 ACEs versus one or more ACEs, and ordinal ACE coding, defined as 0, 1, 2, and 3 or more ACEs.

#### 2.3.3 Academic burnout

Academic burnout was assessed using the student version of the Oldenburg Burnout Inventory. The OLBI has been widely used to assess burnout and includes two core dimensions, disengagement and exhaustion (2, 4). In the present study, the scale comprised 16 items across two dimensions: academic alienation or disengagement and exhaustion. Academic alienation or disengagement reflects psychological distancing from academic work and reduced involvement in learning, whereas exhaustion reflects persistent physical, emotional, and cognitive fatigue related to academic demands.

After reverse scoring the positively worded items, dimension scores were calculated by averaging the corresponding items within each dimension. An overall academic burnout score was then calculated by averaging all 16 items, with higher scores indicating greater academic burnout. The overall score was used as the primary outcome in the regression and mediation analyses because the present study focused on general academic burnout rather than dimension-specific burnout symptoms. Dimension-level descriptive statistics and reliability estimates were reported in **Supplementary Table 4**.

#### 2.3.4 Rumination

Rumination was assessed using the Ruminative Responses Scale (RRS). The RRS is a commonly used measure of repetitive self-focused thinking and includes brooding, reflective pondering, and symptom-focused rumination (14, 15). Symptom rumination reflects repetitive attention to distress-related symptoms. Brooding reflects passive and maladaptive comparisons between one’s current state and unachieved standards. Reflective pondering reflects a more problem-oriented but still repetitive form of self-focused thinking.

The total rumination score was calculated by summing all items, with higher scores indicating a higher tendency toward ruminative thinking. The total score was used in the primary mediation model because the study aimed to examine the overall construct of rumination rather than dimension-specific cognitive processes. Subscale-level descriptive statistics and reliability estimates were reported in **Supplementary Table 4**.

#### 2.3.5 Self-control

Self-control was assessed using the Brief Self-Control Scale (BSCS), which was developed to measure individual differences in the capacity to regulate thoughts, emotions, impulses, and behaviors (17). In the present study, self-control included two dimensions: self-discipline and impulse control. Self-discipline reflects the capacity to persist in goal-directed behavior, whereas impulse control reflects the ability to inhibit immediate urges, distractions, or maladaptive responses. The Chinese version and related adaptations of the BSCS have shown acceptable psychometric properties in Chinese samples (18, 27).

After reverse scoring when applicable, the total self-control score was calculated by summing all items, with higher scores indicating greater self-control. The total score was used in the primary mediation model because the study focused on overall regulatory capacity. Subscale-level descriptive statistics and reliability estimates were reported in **Supplementary Table 4**.

#### 2.3.6 Resilience

Resilience was assessed using the Connor-Davidson Resilience Scale (CD-RISC), which was developed to measure psychological resilience and adaptive coping capacity (20). According to the Chinese three-factor structure, resilience includes tenacity, strength, and optimism (21, 28). Tenacity reflects perseverance under adversity. Strength reflects perceived coping capacity and adaptive competence. Optimism reflects positive expectations for the future.

The total resilience score was calculated by summing all items, with higher scores indicating greater resilience. The total score was used in the primary mediation model because the study examined resilience as an overall adaptive psychological resource. Dimension-level descriptive statistics and reliability estimates were reported in **Supplementary Table 4**.

#### 2.4 Statistical analysis

Data were analyzed using SPSS 27.0 and PROCESS macro version 4.2. Descriptive statistics were used to summarize sample characteristics and study variables. Continuous variables were described using means and standard deviations. Because the ACE cumulative count was highly skewed, its median, interquartile range, skewness, and kurtosis were also reported. Categorical variables were summarized using frequencies and percentages. The internal consistency of multidimensional psychological scales was assessed using Cronbach’s α.

Sex, residence, only-child status, and grade were included as covariates in the adjusted regression and mediation models. Sex was coded as 0 = female and 1 = male. Residence was coded as 0 = urban and 1 = rural. Only-child status was coded as 0 = yes and 1 = no. Grade was dummy-coded with freshmen as the reference group, and four dummy variables were created for sophomores, juniors, seniors, and fifth-year students.

Pearson correlation analyses were used to examine bivariate associations among the primary analytic variables. The primary regression and mediation analyses used the cumulative ACE count, the overall academic burnout score, the total rumination score, the total self-control score, and the total resilience score. In the mediation analyses, unstandardized coefficients were estimated using scale scores, and standardized coefficients were reported to facilitate comparison across paths. Standardized total, direct, and indirect effects were used in the primary interpretation.

Serial mediation was tested using PROCESS Model 6, following the regression-based mediation framework described by Hayes and Rockwood (29). PROCESS was used for the primary serial mediation analysis because the main aim was to estimate the indirect effects of a theoretically informed pathway. Supplementary analyses were conducted to compare alternative mediator orderings. ACEs were specified as the independent variable, academic burnout as the dependent variable, and rumination, self-control, and resilience as statistically linked mediators. Because the data were cross-sectional, the ordering of the mediators was treated as theoretically informed rather than as evidence of temporal or causal sequence. Standard errors and 95% confidence intervals for indirect effects were estimated using 5,000 nonparametric bootstrap resamples, as recommended for testing indirect effects in mediation analysis (30). An indirect effect was considered statistically significant when the 95% bootstrap confidence interval did not include zero.

To assess the robustness of the findings to ACE operationalization, sensitivity analyses were conducted using binary and ordinal ACE indicators. In addition, alternative mediator orderings were examined to evaluate whether the hypothesized ordering was uniquely supported by the data. These analyses included a resilience-first model, a self-control-first model, a rumination-resilience reordering model, a parallel mediation model, and a reverse explanatory model in which academic burnout was specified as a predictor of rumination, self-control, and resilience. For saturated models, global fit indices such as the comparative fit index, Tucker-Lewis index, and root mean square error of approximation were not interpreted because they are not informative when residual degrees of freedom are zero.

Common method bias was examined using Harman’s single-factor test as a preliminary diagnostic procedure. This test was used only as an initial assessment. Because all variables were collected through self-report questionnaires at the same time point, the possibility of common method variance could not be fully excluded (30). Common method bias was examined using Harman’s single-factor test as a preliminary diagnostic procedure.

## 3 Results

### 3.1 Sample characteristics and descriptive statistics

A total of 2,073 questionnaires were collected. After data cleaning, 184 responses were excluded because of multiple submissions from the same IP address, incomplete or prematurely terminated surveys, or patterned or illogical responses. The final analytic sample comprised 1,889 participants. The demographic characteristics and descriptive statistics of the primary study variables are summarized in **Table 1**. To maintain consistency with the primary regression and mediation analyses, ACEs were reported as the cumulative count ranging from 0 to 10. Academic burnout was reported as the overall mean score across the 16 OLBI-S items, whereas rumination, self-control, and resilience were reported as total scores. In the total sample, the mean ACE cumulative count was 0.36 ± 0.90. The mean scores were 1.88 ± 0.49 for academic burnout, 35.03 ± 10.83 for rumination, 25.26 ± 5.24 for self-control, and 72.38 ± 17.91 for resilience.

**Table 1:** Sample characteristics and descriptive statistics of primary study variables (N = 1,889).

Variable	Group	N (%)	ACEs cumulative count, Mean ± SD	Academic Burnout, overall mean score, Mean ± SD	Rumination, total score, Mean ± SD	Self-control, total score, Mean ± SD	Resilience, total score, Mean ± SD
Sex	Male	798 (42.2)	0.34 ± 0.93	1.89 ± 0.52	35.75 ± 11.67	24.45 ± 5.47	71.08 ± 18.89
	Female	1,091 (57.8)	0.36 ± 0.89	1.87 ± 0.47	34.50 ± 10.15	25.86 ± 4.98	73.34 ± 17.11
Residence	Urban	1,046 (55.4)	0.37 ± 0.96	1.86 ± 0.50	35.40 ± 11.76	25.34 ± 5.36	73.49 ± 17.87
	Rural	843 (44.6)	0.34 ± 0.82	1.90 ± 0.48	34.56 ± 9.54	25.16 ± 5.08	71.02 ± 17.88
Only-child status	Yes	888 (47.0)	0.36 ± 0.87	1.83 ± 0.51	34.39 ± 11.13	25.75 ± 5.38	74.05 ± 17.70
	No	1,001 (53.0)	0.35 ± 0.93	1.92 ± 0.47	35.59 ± 10.54	24.83 ± 5.07	70.91 ± 17.98
Grade	Freshmen	332 (17.6)	0.41 ± 0.93	1.95 ± 0.47	37.59 ± 11.19	24.73 ± 4.97	68.94 ± 18.92
	Sophomores	468 (24.8)	0.27 ± 0.84	1.73 ± 0.48	31.69 ± 8.89	26.72 ± 5.35	77.23 ± 16.74
	Juniors	483 (25.6)	0.36 ± 0.81	1.94 ± 0.46	36.40 ± 11.04	24.49 ± 5.10	69.81 ± 16.81
	Seniors	272 (14.4)	0.34 ± 0.76	1.92 ± 0.52	35.21 ± 11.11	25.16 ± 5.26	73.00 ± 18.47
	Fifth-year	334 (17.7)	0.43 ± 1.15	1.88 ± 0.51	35.02 ± 11.32	24.95 ± 5.14	72.23 ± 18.11
Total sample		1,889 (100.0)	0.36 ± 0.90	1.88 ± 0.49	35.03 ± 10.83	25.26 ± 5.24	72.38 ± 17.91

ACEs, adverse childhood experiences; SD, standard deviation. ACEs were assessed as a cumulative count ranging from 0 to 10. Academic burnout was calculated as the overall mean score across the 16 OLBI-S items, ranging from 1 to 4. Rumination, self-control, and resilience are presented as total scores. The theoretical score ranges were 22–88 for rumination, 7–35 for self-control, and 25–125 for resilience. Percentages were calculated within each demographic characteristic.

### 3.2 Measurement properties, ACE distribution, and correlations

Because academic burnout, rumination, self-control, and resilience are multidimensional measures, dimension-level descriptive statistics and reliability estimates are provided in **Supplementary Table 1**. The cumulative ACE count was highly right-skewed (median = 0, interquartile range = 0–0), with 76.7% of participants reporting no ACE exposure. Sensitivity analyses using binary (0 vs ≥1) and ordinal (0, 1, 2, ≥3) ACE indicators indicated that the main results were robust to alternative ACE operationalizations (**Supplementary Tables 2a, b**). The distribution of ACE exposure by domain and item is summarized in **Supplementary Table 3**, with household dysfunction being the most frequently reported domain, followed by neglect and abuse. Percentages cannot be summed across categories because participants could report multiple ACEs. Pearson correlation analyses (**Table 2**) indicated that higher ACE exposure was associated with greater academic burnout and rumination, and lower self-control and resilience. Academic burnout was positively correlated with rumination and negatively correlated with self-control and resilience, whereas self-control and resilience were positively correlated with each other.

**Table 2:** Pearson correlation matrix among variables.

Variable	ACEs	Academic burnout	Rumination	Self-control	Resilience
ACEs	1				
Academic Burnout	0.187^**^	1			
Rumination	0.226^**^	0.519^**^	1		
Self-control	-0.154^**^	-0.621^**^	-0.473^**^	1	
Resilience	-0.211^**^	-0.686^**^	-0.494^**^	0.546^**^	1

N = 1,889. ACEs = Adverse Childhood Experiences. **p < 0.01 (two-tailed test).

### 3.3 Regression and serial mediation analyses

**Figure 2:**
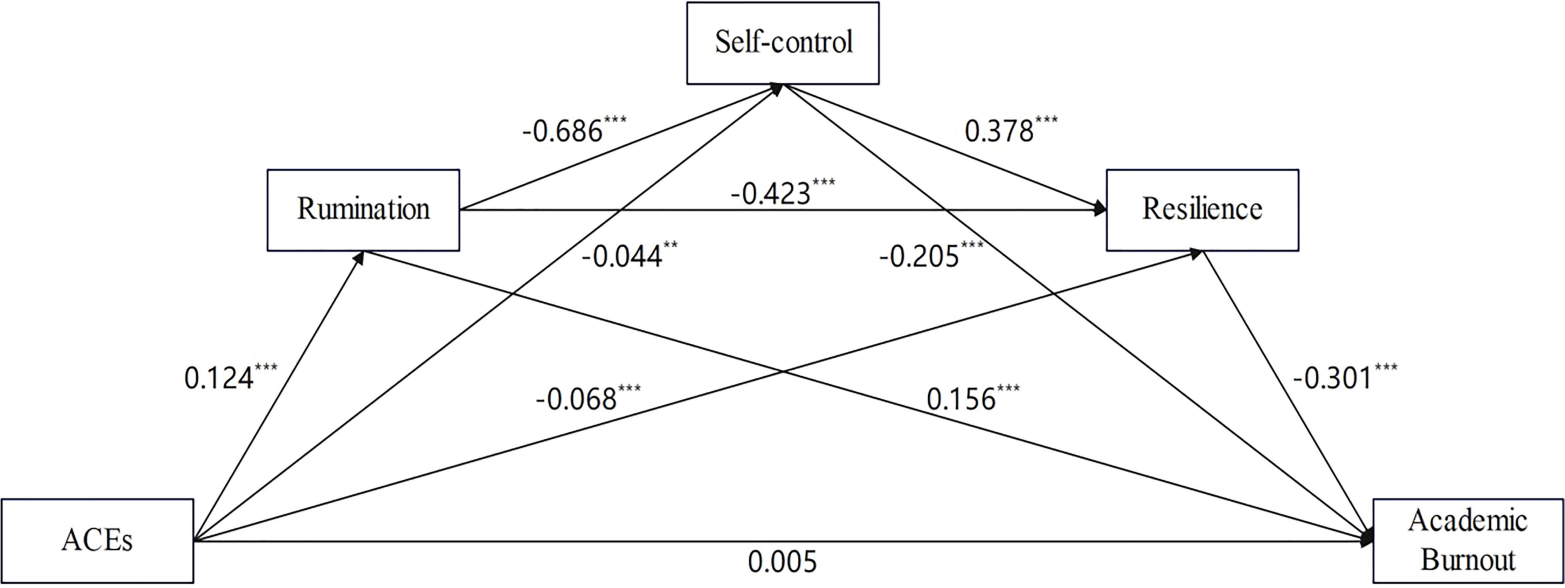
Standardized path coefficients for the final serial mediation model. *p < 0.05, **p < 0.01, ***p < 0.001. Ind1: ACEs → Rumination → Academic Burnout; Ind2: ACEs → Self-control → Academic Burnout; Ind3: ACEs → Resilience → Academic Burnout; Ind4: ACEs → Rumination → Self-control → Academic Burnout; Ind5: ACEs → Rumination → Resilience → Academic Burnout; Ind6: ACEs → Self-control → Resilience → Academic Burnout; Ind7: ACEs → Rumination → Self-control → Resilience → Academic Burnout.

Hierarchical regression analyses predicting academic burnout from ACEs are presented in **Table 3**. In the unadjusted model, the cumulative ACE count was positively associated with academic burnout. After adjusting for sex, residence, only-child status, and dummy-coded grade, ACEs remained significantly associated with academic burnout. Among the covariates, only-child status and sophomore grade were significantly related to burnout, whereas sex, residence, and other grade levels were not. Variance inflation factor values ranged from 1.00 to 1.86, indicating no serious multicollinearity. The corresponding path diagram with standardized coefficients is presented in **Figure 2**.

**Table 3:** Hierarchical regression analysis predicting academic burnout from ACEs.

Variable		B	SE	t	β	R2	Adjusted R²	F	p
Model 1	constant	1.840	0.012	153.985***		0.035	0.034	68.115	<0.001
	ACEs	1.017	0.123	8.253***	0.187				<0.001
Model 2	constant	1.780	0.058	30.776***		0.074	0.070	11.450	<0.001
	Sex	-0.022	0.022	-0.993	-0.022				0.321
	Residence	0.018	0.024	0.742	0.092				0.458
	Only child status	0.090	0.024	3.843***	0.092				<0.001
	Grade Sophomore (vs freshman)	-0.210	0.034	-6.167***	-0.185				<0.001
	Grade Junior (vs freshman)	0.001	0.034	0.018	0.001				0.986
	Senior (vs freshman)	-0.023	0.039	-0.604	-0.017				0.546
	Grade Fifth-year (vs freshman)	-0.064	0.037	-1.730	-0.05				0.084
	ACEs	0.977	0.121	8.062***	0.179				<0.001
ΔR2						0.039*			
ΔF								11.450***	
VIF		1.00-1.86							

In the analyses presented in this paper, *** denotes p < 0.001 (highly significant) and is used to indicate strong effects of core predictor variables such as ACEs and only-child status; * denotes p < 0.05 (significant) and is used to mark significant improvements in the model’s ΔR^2^.

**Table 4** presents the standardized path coefficients for the serial mediation model. ACEs were positively associated with rumination and negatively associated with self-control and resilience. Rumination, in turn, was negatively associated with both self-control and resilience, and self-control was positively associated with resilience. In predicting academic burnout, rumination was positively associated, whereas self-control and resilience were negatively associated with burnout. In this study, total scores of the multidimensional scales were used for the primary analyses to capture the overall constructs of academic burnout, rumination, self-control, and resilience, while dimension-level descriptive statistics and reliability estimates are provided in **Supplementary Table 1** for transparency.

**Table 4:** Path coefficients for the serial mediation model.

	Rumination			Self-control			Resilience			Academic burnout			Total		
	B	SE	β	B	SE	β	B	SE	β	B	SE	β	B	SE	β
constant	1.69	0.057		4.605	0.096		2.351	0.124		3.116	0.077		1.780	0.058	
ACEs	1.19	0.120	0.218	-0.436	0.170	-0.053	-0.680	0.148	-0.086	0.058	0.085	0.011	0.977	0.121	0.179
Rumination				-0.666	0.032	-0.438	-0.411	0.031	-0.283	0.153	0.018	0.153			
Self-control							0.373	0.020	0.390	-0.204	0.012	-0.310			
Resilience										-0.300	0.013	-0.437			
Sex	-0.052	0.022	-0.053	0.168	0.031	0.111	-0.006	0.027	-0.004	0.054	0.015	0.055	-0.022	0.022	-0.022
Residence	-0.062	0.023	-0.062	-0.023	0.033	-0.015	-0.101	0.028	-0.070	0.010	0.016	0.010	0.018	0.024	0.018
Only child status	0.087	0.023	0.089	-0.100	0.032	-0.067	-0.021	0.028	-0.015	0.010	0.016	0.010	0.090	0.024	0.092
Sophomore vs freshman	-0.250	0.034	-0.219	0.106	0.047	0.061	0.112	0.041	0.068	-0.022	0.023	-0.019	-0.210	0.034	-0.185
Junior vs freshman	-0.046	0.034	-0.041	-0.050	0.047	-0.029	0.030	0.041	0.019	0.016	0.023	0.014	0.001	0.034	0.001
Senior vs freshman	-0.093	0.038	-0.067	-0.016	0.053	-0.007	0.098	0.046	0.048	0.046	0.026	0.033	-0.024	0.039	-0.017
Fifth-year vs freshman	-0.106	0.037	-0.082	-0.060	0.051	-0.031	0.081	0.044	0.043	-0.007	0.025	-0.006	-0.064	0.037	-0.050
R2	0.095			0.249			0.386			0.579			0.074		
F	24.64*			69.32*			118.09*			234.75*			18.86*		

The asterisk * superscripted to the F‑statistic values indicates that the overall test of the regression model (i.e., the F‑test) is statistically significant.

The total, direct, and indirect effects of ACEs on academic burnout are shown in **Table 5**. The total effect of ACEs on academic burnout was significant. After including rumination, self-control, and resilience in the model, the direct effect of ACEs was no longer statistically significant, whereas the total indirect effect remained significant. Specifically, ACEs were statistically associated with academic burnout indirectly through higher rumination, lower self-control, and lower resilience. In addition, several serial indirect pathways were significant, indicating that ACEs were linked to burnout sequentially via rumination and self-control, via rumination and resilience, via self-control and resilience, and through a combined pathway involving rumination, self-control, and resilience. All seven specific indirect effects had 95% bootstrap confidence intervals that excluded zero, supporting the presence of multiple statistically significant indirect associations among ACEs, the psychological variables, and academic burnout.

**Table 5:** Total, direct, and indirect effects in the association between ACEs and academic burnout. .

Effect/pathway	Effect	Boot SE	95% Boot CI lower	95% Boot CI upper	Interpretation
Total effect	0.1810	0.0298	0.1253	0.2436	Significant
Direct effect	0.0120	0.0225	-0.0287	0.0592	NS
Total indirect effect	0.1690	0.0176	0.1348	0.2042	Significant
ACEs → Rumination → Burnout	0.0335	0.0077	0.0196	0.0497	Significant
ACEs → Self-control → Burnout	0.0163	0.0069	0.0021	0.0293	Significant
ACEs → Resilience → Burnout	0.0363	0.0158	0.0050	0.0668	Significant
ACEs → Rumination → Self-control → Burnout	0.0300	0.0067	0.0180	0.0441	Significant
ACEs → Rumination → Resilience → Burnout	0.0275	0.0068	0.0155	0.0421	Significant
ACEs → Self-control → Resilience → Burnout	0.0090	0.0039	0.0011	0.0165	Significant
ACEs → Rumination → Self-control → Resilience → Burnout	0.0165	0.0037	0.0099	0.0244	Significant

Estimates are standardized path coefficients from the three-mediator serial mediation model (Model 0): ACEs → Rumination → Self-control → Resilience → Academic burnout. Boot SE and 95% Boot CIs based on 5,000 bootstrap resamples. Covariates: sex, residence, only-child status, and dummy-coded grade (reference = freshman). Significance is judged solely by whether the 95% Boot CI excludes zero, in line with the reviewer’s recommendation. Proportion-mediated values are not reported as they can be unstable and may overstate causal claims in cross-sectional data.

### 3.4 Sensitivity analyses and alternative model comparisons

Sensitivity analyses were conducted using three ACE operationalizations: continuous cumulative count, binary ACE exposure, and ordinal ACE exposure coded as 0, 1, 2, and 3 or more ACEs (**Supplementary Table 2b**). In all three models, the total effect of ACEs on academic burnout was statistically significant. After rumination, self-control, and resilience were included in the models, the direct effect of ACEs on academic burnout was not statistically significant, whereas the total indirect effect was statistically significant.

Alternative mediator orderings were tested and are presented in **Supplementary Tables 4** and **S5**. The hypothesized serial mediation model and several alternative serial mediation models were saturated. The parallel mediation model showed poorer model fit. The reverse explanatory model, in which academic burnout was specified as a predictor of rumination, self-control, and resilience, showed acceptable model fit. The corresponding indirect effects and path estimates are summarized in **Supplementary Table 5**.

## 4 Discussion

This study examined the association between adverse childhood experiences and academic burnout among students at a Chinese traditional medicine university, with particular attention to rumination, self-control, and resilience as statistically linked psychological variables. Three main findings emerged. First, cumulative ACE exposure was positively associated with academic burnout in this sample. Second, rumination, self-control, and resilience each contributed to the ACEs–burnout association through significant indirect pathways. Third, the overall pattern remained broadly consistent across alternative ACE codings, whereas several alternative mediator orderings were also statistically plausible. Taken together, these findings support a cautious interpretation: ACEs were associated with higher burnout, and cognitive-regulatory and resilience-related variables were related to this association, but the present cross-sectional data do not identify a single definitive temporal pathway.

The association between ACEs and academic burnout is consistent with prior evidence indicating that early adversity is linked to poorer psychological adjustment, greater vulnerability to distress, and difficulties in educational functioning among young adults (8–11). In medical education, students are exposed to intensive coursework, repeated assessment, and prolonged training demands, which may amplify the impact of limited psychological resources (1, 5, 6). From a developmental perspective, ACEs may reflect early exposure to unstable or unsupportive environments that shape later stress appraisal and coping expectations (9, 10). Our findings are therefore compatible with the view that early adversity is associated with greater susceptibility to academic burnout, while still stopping short of implying that ACEs directly or inevitably cause burnout.

The distribution of ACE exposure in this sample deserves attention. Most participants reported no ACE exposure, and the cumulative ACE count was strongly right-skewed. This pattern may reflect a relatively low level of reported adversity, but it may also be influenced by recall limitations, social desirability, or reluctance to disclose family-related adversity (9, 11). In Chinese student samples, family privacy and stigma may shape how adverse experiences are remembered and reported (11, 31, 32). Because ACE exposure was concentrated at the lower end of the distribution, we conducted sensitivity analyses using binary and ordinal operationalizations. The main pattern of findings was preserved, suggesting that the observed associations were not dependent on treating ACEs as a continuous count. Nevertheless, the skewed distribution remains an important limitation when interpreting the magnitude and stability of the indirect effects.

Rumination emerged as an important correlate in the ACEs–burnout association. Students with greater ACE exposure reported higher rumination, and rumination was positively associated with academic burnout. This aligns with evidence that repetitive negative thinking can prolong distress, reduce cognitive flexibility, and interfere with adaptive problem solving (14, 15). In academic contexts, rumination may keep students focused on distressing experiences rather than current tasks, thereby contributing to exhaustion and disengagement (16, 33). Prior work in university students has likewise shown that rumination can mediate the association between academic stress and burnout (16). However, the present cross-sectional design does not permit conclusions about directionality. It remains possible that students with greater burnout become more prone to repetitive negative thinking, particularly when they perceive academic demands as overwhelming.

Self-control also showed a meaningful association with academic burnout. Students with greater ACE exposure tended to report lower self-control, and lower self-control was associated with higher burnout. This pattern is consistent with the view that self-control supports sustained academic engagement, attention regulation, and emotional restraint (17, 18). Prior research has linked self-control and self-regulatory fatigue to student burnout and academic functioning (22, 34, 35). Importantly, self-control should not be interpreted as a fixed personal deficit. Rather, it is a context-sensitive regulatory capacity that may be influenced by stress, fatigue, and environmental demands. Accordingly, the present findings indicate that self-control is statistically related to the ACEs–burnout association, but they do not establish that ACEs directly depleted self-control over time.

Resilience was one of the strongest correlates of burnout in the present study. Higher resilience was associated with lower academic burnout, consistent with prior work in medical students and other university populations showing inverse associations between resilience and burnout (20, 21). Similar findings have also been reported for psychological distress and academic strain (23, 36). In the present study, resilience was conceptualized as a multidimensional adaptive resource including tenacity, strength, and optimism (28). These dimensions may help students maintain motivation and recover from academic setbacks, particularly in educational settings characterized by prolonged demands and limited recovery opportunities (37, 38). At the same time, resilience may also reflect accumulated adaptive experiences and coping success over time, which means that moderation or reciprocal associations with burnout remain plausible (23, 37).

The supplementary analyses are especially informative for interpreting the serial mediation model. Although the hypothesized ordering of rumination, self-control, and resilience was theoretically reasonable, alternative models were also statistically plausible. This is not unexpected, because cross-sectional path models primarily capture shared covariance rather than temporal sequence (29, 30). Some serial specifications were saturated, so global fit indices could not meaningfully distinguish among them (29). The parallel mediation model showed weaker support than the serial specifications, suggesting that these variables should not be treated merely as unrelated parallel correlates. However, the reverse explanatory model remained statistically compatible with the observed data, indicating that burnout-related pathways are also plausible (16, 22). Together, these findings highlight a key methodological issue: cross-sectional path analysis can identify statistically tenable models, but it cannot determine which model is temporally correct (17, 29, 30).

From a theoretical standpoint, the findings are broadly compatible with Conservation of Resources theory, but they do not validate a single resource-loss sequence (24, 25). COR theory suggests that stress arises when valued resources are threatened or depleted, and that individuals attempt to conserve or rebuild resources under adversity (24). In this framework, ACEs may reflect early resource loss, rumination may represent cognitively costly negative processing, self-control may capture current regulatory capacity, and resilience may reflect broader adaptive resources (25, 39). Yet COR theory does not require a fixed ordering of these variables. Low self-control may increase rumination, lower resilience may sustain negative thinking, and burnout may in turn erode adaptive resources (17, 22, 35, 40). Thus, the theoretical contribution of this study lies in showing that these constructs are interwoven in a pattern consistent with resource-based explanations of burnout.

The present findings may have practical implications for student mental health support in traditional medicine education. Students with a history of childhood adversity may benefit from confidential, non-stigmatizing screening and support (9, 11, 13). Interventions that target rumination, self-regulation, and resilience may be useful components of burnout prevention (41–43). However, because intervention effects were not tested here, these implications remain preliminary.

Several limitations should be noted. First, the cross-sectional design precludes conclusions about temporal ordering or causality (16, 29). Second, all variables were self-reported at the same time point, which may introduce recall bias and common method variance despite the preliminary Harman test (30). Third, ACE exposure was highly skewed, and although sensitivity analyses suggested robustness across alternative coding schemes, restricted variability may affect effect-size stability (11, 39). Fourth, the sample was drawn from a single traditional Chinese medicine university in Northeast China, which limits generalizability. Fifth, the study did not measure biological, neurocognitive, or contextual support variables, so mechanistic interpretation should remain tentative (35, 43).

In conclusion, this study found that cumulative ACE exposure was associated with academic burnout among students at a Chinese traditional medicine university. Rumination, self-control, and resilience were each statistically related to this association, and the overall pattern was robust to alternative ACE operationalizations. At the same time, alternative mediator sequences remained plausible, underscoring the need for cautious interpretation. Longitudinal and intervention studies are needed to clarify temporal ordering and to determine whether modifying these psychological factors can reduce academic burnout.

## Data Availability

The original contributions presented in the study are included in the article/**Supplementary Material**. Further inquiries can be directed to the corresponding author.

## References

[1] Almutairi H Alsubaiei A Abduljawad S Alshatti A Fekih-Romdhane F Husni M . Prevalence of burnout in medical students: a systematic review and meta-analysis. Int J Soc Psychiatry. (2022) 68:1157–70. doi: 10.1177/00207640221106691 35775726

[2] Reis D Xanthopoulou D Tsaousis I . Measuring job and academic burnout with the Oldenburg Burnout Inventory (OLBI): factorial invariance across samples and countries. Burnout Res. (2015) 2:8–18. doi: 10.1016/j.burn.2014.11.001 38826717

[3] Smith A Ellison J Bogardus J Gleeson P . Reliability and validity of the student version of the Oldenburg Burnout Inventory in physical therapist students. J Phys Ther Educ. (2022) 36(3):205–9. doi: 10.1097/JTE.0000000000000222 42290303

[4] Demerouti E Mostert K Bakker AB . Burnout and work engagement: a thorough investigation of the independency of both constructs. J Occup Health Psychol. (2010) 15:209–22. doi: 10.1037/a0019408 20604629

[5] Kiss H Piko BF . Risk and protective factors of student burnout among medical students: a multivariate analysis. BMC Med Educ. (2025) 25:386. doi: 10.1186/s12909-025-06956-8 40089769 PMC11910849

[6] Di Vincenzo M Arsenio E Della Rocca B Rosa A Tretola L Toricco R . Is there a burnout epidemic among medical students? Results from a systematic review. Med (Kaunas). (2024) 60(4):575. doi: 10.3390/medicina60040575 38674221 PMC11052230

[7] Faghihzadeh E Eghtesad A Fawad M Xu X . Exploring connections between mental health, burnout, and academic factors among medical students at an Iranian university: cross-sectional questionnaire study. JMIR Med Educ. (2025) 11:e58008. doi: 10.2196/58008 40372966 PMC12097282

[8] Felitti VJ Anda RF Nordenberg D Williamson DF Spitz AM Edwards V . Relationship of childhood abuse and household dysfunction to many of the leading causes of death in adults. The Adverse Childhood Experiences (ACE) Study. Am J Prev Med. (1998) 14:245–58. doi: 10.1016/s0749-3797(98)00017-8 9635069

[9] Kalmakis KA Chandler GE . Adverse childhood experiences: towards a clear conceptual meaning. J Adv Nurs. (2014) 70:1489–501. doi: 10.1111/jan.12329 24329930

[10] Hughes K Bellis MA Hardcastle KA Sethi D Butchart A Mikton C . The effect of multiple adverse childhood experiences on health: a systematic review and meta-analysis. Lancet Public Health. (2017) 2:e356–66. doi: 10.1016/S2468-2667(17)30118-4 29253477

[11] Chen W Yu Z Wang L Gross D . Examining childhood adversities in Chinese health science students using the simplified Chinese version of the Adverse Childhood Experiences-International Questionnaire (SC-ACE-IQ). Advers Resil Sci. (2022) 3:335–46. doi: 10.1007/s42844-022-00076-8 36278243 PMC9580443

[12] Yang P Robles-Ramamurthy B Plastino KA . Associations between adverse childhood experiences and medical students' interest in careers: a single-setting study. Front Psychiatry. (2025) 16:1483871. doi: 10.3389/fpsyt.2025.1483871 40104329 PMC11913809

[13] Kara Esen B Tugcu UE Inan K Can G . Effects of adverse childhood experiences on health behaviors among medical students: a cross-sectional study. Med (Baltimore). (2025) 104:e43259. doi: 10.1097/MD.0000000000043259 40660598 PMC12262963

[14] Nolen-Hoeksema S Morrow J . A prospective study of depression and posttraumatic stress symptoms after a natural disaster: the 1989 Loma Prieta Earthquake. J Pers Soc Psychol. (1991) 61:115–21. doi: 10.1037//0022-3514.61.1.115 1890582

[15] Treynor W Gonzalez R Nolen-Hoeksema S . Rumination reconsidered: a psychometric analysis. Cogn Ther Res. (2003) 27:247–59. doi: 10.1023/A:1023910315561 41886696

[16] Zuo X Zhao L Li Y He W Yu C Wang Z . Psychological mechanisms of English academic stress and academic burnout: the mediating role of rumination and moderating effect of neuroticism. Front Psychol. (2024) 15:1309210. doi: 10.3389/fpsyg.2024.1309210 38328384 PMC10847527

[17] Tangney JP Baumeister RF Boone AL . High self-control predicts good adjustment, less pathology, better grades, and interpersonal success. J Pers. (2004) 72:271–324. doi: 10.1111/j.0022-3506.2004.00263.x 15016066

[18] Morean ME DeMartini KS Leeman RF Pearlson GD Anticevic A Krishnan-Sarin S . Psychometrically improved, abbreviated versions of three classic measures of impulsivity and self-control. Psychol Assess. (2014) 26:1003–20. doi: 10.1037/pas0000003 24885848 PMC4152397

[19] Papanikolopoulos P Mastrotheodoros S Papanikolopoulou S Parlapani E Kaprinis SG . The brief self-control scale: dimensionality and psychometric properties in Greek young adults. Eur J Dev Psychol. (2022) 19:925–37. doi: 10.1080/17405629.2021.1964070 37339054

[20] Connor KM Davidson JR . Development of a new resilience scale: the Connor-Davidson Resilience Scale (CD-RISC). Depress Anxiety. (2003) 18:76–82. doi: 10.1002/da.10113 12964174

[21] Wu L Tan Y Liu Y . Factor structure and psychometric evaluation of the Connor-Davidson resilience scale in a new employee population of China. BMC Psychiatry. (2017) 17:49. doi: 10.1186/s12888-017-1219-0 28152997 PMC5290619

[22] Zhang J Meng J Wen X . The relationship between stress and academic burnout in college students: evidence from longitudinal data on indirect effects. Front Psychol. (2025) 16:1517920. doi: 10.3389/fpsyg.2025.1517920 40491945 PMC12146318

[23] Alhowaymel F . The relationship between adverse childhood experiences and resilience among college students in Saudi Arabia: a cross-sectional study. Sci Rep. (2025) 15:34239. doi: 10.1038/s41598-025-16250-8 41034301 PMC12489094

[24] Hobfoll S Halbesleben J Neveu J-P Westman M . Conservation of resources in the organizational context: the reality of resources and their consequences. Annu Rev Organizational Psychol Organizational Behav. (2018) 5:103–28. doi: 10.1146/annurev-orgpsych-032117-104640 41139587

[25] Hobfoll SE . Conservation of Resources Theory: its implication for stress, health, and resilience. In: Folkman S , editor. The Oxford Handbook of Stress, Health, and Coping. Oxford University Press (2012). p. 127–147.

[26] Demerouti E . Job demands-resources and conservation of resources theories: how do they help to explain employee well-being and future job design? J Business Res. (2025) 192:115296. doi: 10.1016/j.jbusres.2025.115296 38826717

[27] Zhang S Fang Z Qi X Yuan Y Xiao K Bian Z . Validation and adaptation of the Brief Self-Control Scale for internet use among Chinese adolescents: factor structure, reliability, validity, and measurement invariance across gender and age. Acta Psychol (Amst). (2025) 254:104849. doi: 10.1016/j.actpsy.2025.104849 40020288

[28] Yu NX Zhang J . Factor analysis and psychometric evaluation of the Connor-Davidson Resilience Scale (CD-RISC) with Chinese people. Soc Behav Personality: Int J. (2007) 35:19–30. doi: 10.2224/sbp.2007.35.1.19

[29] Hayes AF Rockwood NJ . Regression-based statistical mediation and moderation analysis in clinical research: observations, recommendations, and implementation. Behav Res Ther. (2017) 98:39–57. doi: 10.1016/j.brat.2016.11.001 27865431

[30] Podsakoff PM MacKenzie SB Lee JY Podsakoff NP . Common method biases in behavioral research: a critical review of the literature and recommended remedies. J Appl Psychol. (2003) 88:879–903. doi: 10.1037/0021-9010.88.5.879 14516251

[31] Williamson L Daniel SS Carter J Ridenhour A Pulgar CA Gay Y . Negative effects of adverse childhood experiences and absence of positive childhood experiences on healthcare employees: survey findings built on 10 years of trauma-informed development. Front Public Health. (2024) 12:1494587. doi: 10.3389/fpubh.2024.1494587 39835305 PMC11743665

[32] Haslam SK Hamilton-Hinch B Torres S Munroe A Grant T Gilbert R . Adverse childhood experiences, maladaptive coping behaviours and protective factors in undergraduate students: a cross-sectional study. J Am Coll Health. (2025) 73:3421–32. doi: 10.1080/07448481.2024.2412072 39383094

[33] Ijaz T Khalid A . Perfectionism and academic burnout: the mediating role of worry and depressive rumination in university students. Pakistan J psychol Res. (2020) 35:473–92. doi: 10.33824/pjpr.2020.35.3.25

[34] Baumeister RF Bratslavsky E Muraven M Tice DM . Ego depletion: is the active self a limited resource? J Pers Soc Psychol. (1998) 74:1252–65. doi: 10.1037//0022-3514.74.5.1252 9599441

[35] Troll ES Friese M Loschelder DD . Do we fail to exert self-control because we lack resources or motivation? Competing theories to explain a debated phenomenon. Br J Soc Psychol. (2023) 62:782–805. doi: 10.1111/bjso.12594 36329599

[36] Fletcher D Sarkar M . Psychological resilience: a review and critique of definitions, concepts, and theory. Eur Psychol. (2013) 18:12–23. doi: 10.1027/1016-9040/a000124

[37] Kalisch R Baker DG Basten U Boks MP Bonanno GA Brummelman E . The resilience framework as a strategy to combat stress-related disorders. Nat Hum Behav. (2017) 1:784–90. doi: 10.1038/s41562-017-0200-8 31024125

[38] Joyce S Shand F Tighe J Laurent SJ Bryant RA Harvey SB . Road to resilience: a systematic review and meta-analysis of resilience training programmes and interventions. BMJ Open. (2018) 8:e017858. doi: 10.1136/bmjopen-2017-017858 29903782 PMC6009510

[39] Campbell-Sills L Forde DR Stein MB . Demographic and childhood environmental predictors of resilience in a community sample. J Psychiatr Res. (2009) 43:1007–12. doi: 10.1016/j.jpsychires.2009.01.013 19264325

[40] Jin M Ding L Fan J Sheng X Luo B Hang R . Moderating role of resilience between depression and stress response of vocational middle school students during the COVID-19 pandemic. Front Psychiatry. (2022) 13:904592. doi: 10.3389/fpsyt.2022.904592 35845471 PMC9283898

[41] Zheng Y Gu X Jiang M Zeng X . How might mindfulness-based interventions reduce job burnout? Testing a potential self-regulation model with a randomized controlled trial. Mindfulness. (2022) 13:1–16. doi: 10.1007/s12671-022-01927-2 34539929

[42] Banerjee Y Akhras A Khamis AH Alsheikh-Ali A Davis D . Investigating the relationship between resilience, stress-coping strategies, and learning approaches to predict academic performance in undergraduate medical students: protocol for a proof-of-concept study. JMIR Res Protoc. (2019) 8:e14677. doi: 10.2196/14677 31538947 PMC6754686

[43] Dang J Xiao S Mao L Liu X Baumert A Bonneterre S . Revisiting ego depletion: evidence from multi-lab collaborations. J Pacific Rim Psychol. (2025) 19:1–10. doi: 10.1177/18344909251386084

