# Supplementary material for: Adverse childhood experiences and academic burnout among Chinese traditional medicine students: the serial mediating role of rumination, self-control, and resilience
Source: Front Psychiatry. 2026 Jul 9;17:1829698. doi: 10.3389/fpsyt.2026.1829698 (PMC13391914; doi:10.3389/fpsyt.2026.1829698)
Supplement: Supplementary file 1 [file Table1.docx]

**Supplementary Table S1. Descriptive statistics and reliability of multidimensional psychological scales.**

| **Construct** | **Scale score / dimension** | **No. of items** | **Theoretical range** | **Observed range** | **Mean ± SD** | **Cronbach's α** |
| --- | --- | --- | --- | --- | --- | --- |
| Academic burnout | Academic burnout, overall score | 16 | 1–4 | 1.00–4.00 | 1.876 ± 0.492 | 0.915 |
| Academic burnout | Academic alienation | 8 | 1–4 | 1.00–4.00 | 1.884 ± 0.486 | 0.803 |
| Academic burnout | Exhaustion | 8 | 1–4 | 1.00–4.00 | 1.869 ± 0.552 | 0.891 |
| Rumination (RRS) | Rumination, total score | 22 | 22–88 | 22–88 | 35.03 ± 10.83 | 0.949 |
| Rumination (RRS) | Symptom rumination | 12 | 12–48 | 12–48 | 17.81 ± 6.01 | 0.939 |
| Rumination (RRS) | Brooding | 5 | 5–20 | 5–20 | 8.71 ± 2.89 | 0.811 |
| Rumination (RRS) | Reflective pondering | 5 | 5–20 | 5–20 | 8.50 ± 2.83 | 0.765 |
| Self-control (BSCS) | Total BSCS score | 7 | 7–35 | 7–35 | 25.26 ± 5.24 | 0.807 |
| Self-control (BSCS) | Self-discipline | 3 | 3–15 | 3–15 | 11.42 ± 2.68 | 0.882 |
| Self-control (BSCS) | Impulse control | 4 | 4–20 | 4–20 | 13.85 ± 3.74 | 0.807 |
| Resilience (CD-RISC) | Total CD-RISC score | 25 | 0–100 | 0–100 | 72.38 ± 17.91 | 0.967 |
| Resilience (CD-RISC) | Tenacity | 12 | 0–48 | 0–48 | 35.85 ± 9.15 | 0.957 |
| Resilience (CD-RISC) | Strength | 9 | 0–36 | 0–36 | 25.70 ± 6.51 | 0.901 |
| Resilience (CD-RISC) | Optimism | 4 | 0–16 | 0–16 | 10.83 ± 2.94 | 0.668 |

*Note. RRS subscales (Treynor et al., 2003): Brooding (5 items), Reflective pondering (5 items), Symptom rumination (remaining 12 items). BSCS subscales (Tan & Guo): Self-discipline (items 1–3, reverse-scored when applicable), Impulse control (items 4–7). CD-RISC subscales (Yu & Zhang, 2007, Chinese three-factor structure): Tenacity (12 items), Strength (9 items), Optimism (4 items). Total score was used in the primary mediation model; dimension-level descriptive statistics are reported here for transparency.*

**Supplementary Table S2a. Distribution of cumulative ACEs.**

| **ACEs measure** | **Category / statistic** | **N** | **%** |
| --- | --- | --- | --- |
| ACE count | 0 | 1449 | 76.70% |
| ACE count | 1 | 325 | 17.20% |
| ACE count | 2 | 70 | 3.70% |
| ACE count | ≥3 | 45 | 2.40% |
| Binary ACE exposure | 0 ACEs | 1449 | 76.70% |
| Binary ACE exposure | ≥1 ACE | 440 | 23.30% |
| ACE count | Mean ± SD | 0.36 | — |
| ACE count | Median (IQR) | 0 | — |
| ACE count | Skewness | 5.25 | — |
| ACE count | Kurtosis (excess) | 41.10 | — |

*Note. ACEs cumulative count (0–10) was used as the independent variable in the primary regression and mediation analyses. Binary and ordinal ACE codings were used in sensitivity analyses. The high skewness and kurtosis indicate a strongly right-skewed distribution.*

**Supplementary Table S2b. Sensitivity analysis of ACE operationalization.**

| ACEs operationalization | B (ACEs → Academic Burnout) | SE | p value | Total indirect | 95% Boot CI | Direct effect | 95% Boot CI | Interpretation |
| --- | --- | --- | --- | --- | --- | --- | --- | --- |
| Continuous ACE count (0–10) | 0.098 | 0.012 | < 0.001 | 0.169 | (0.135, 0.204) | 0.012 | (-0.029, 0.059) | Significant total indirect; non-significant direct |
| Binary ACE exposure (0 vs ≥1) | 0.164 | 0.026 | < 0.001 | 0.123 | (0.089, 0.156) | 0.019 | (-0.010, 0.049) | Pattern preserved under dichotomization |
| Ordinal ACE exposure (0/1/2/≥3) | 0.136 | 0.017 | < 0.001 | 0.158 | (0.125, 0.191) | 0.024 | (-0.013, 0.063) | Pattern preserved under categorization |

*Note. B = unstandardized regression coefficient (adjusted for sex, residence, only-child status, and grade dummy variables). Total indirect and direct effects are standardized estimates from the four-mediator serial mediation model with 5,000 bootstrap resamples.*

**Supplementary Table S3. Distribution of adverse childhood experiences by domain and item.**

| **ACE domain / item** | **n** | **% of total sample (N = 1,889)** | **% among ACE-exposed (n = 440)** |
| --- | --- | --- | --- |
| Any ACE exposure | 440 | 23.3 | 100 |
| Abuse (any) | 93 | 4.9 | 21.1 |
| Abuse (emotional) | 61 | 3.2 | 13.9 |
| Abuse (physical) | 45 | 2.4 | 10.2 |
| Abuse (sexual) | 28 | 1.5 | 6.4 |
| Neglect (any) | 179 | 9.5 | 40.7 |
| Neglect (emotional) | 173 | 9.2 | 39.3 |
| Neglect (physical) | 23 | 1.2 | 5.2 |
| Household dysfunction (any) | **276** | **14.6** | **62.7** |
| Household dysfunction (parental separation/divorce) | 236 | 12.5 | 53.6 |
| Household dysfunction (domestic violence) | 30 | 1.6 | 6.8 |
| Household dysfunction (substance abuse) | 14 | 0.7 | 3.2 |
| Household dysfunction (mental illness) | 35 | 1.9 | 8 |
| Household dysfunction (incarcerated household member) | 27 | 1.4 | 6.1 |

*Note. ACE domains and individual items are not mutually exclusive; percentages cannot be summed because the same participant may have reported multiple ACEs.*

**Supplementary Table S4. Comparison of the hypothesized and alternative path models: fit indices.**

| **Model** | **Key model structure** | **χ² / df** | **CFI** | **TLI** | **RMSEA** | **AIC** | **BIC** | **Interpretation** |
| --- | --- | --- | --- | --- | --- | --- | --- | --- |
| **Model 0** | ACEs → Rum → SC → Res → Burnout | — | — | — | — | 84.0 | 316.8 | Primary hypothesized model (saturated) |
| **Model A** | ACEs → Res → Rum → Burnout | — | — | — | — | 60.0 | 226.3 | Resilience-first ordering (saturated) |
| **Model B** | ACEs → SC → Rum → Burnout | — | — | — | — | 60.0 | 226.3 | Self-control-first ordering (saturated) |
| **Model C** | ACEs → Rum → Res → Burnout | — | — | — | — | 60.0 | 226.3 | Rum-resilience reordering (saturated) |
| **Model D** | ACEs → {Rum, SC, Res} → Burnout | 1145.191/39 | 0.782 | 0.587 | 0.123 | 76.8 | 293.0 | Parallel mediation |
| **Model E** | Burnout → {Rum, SC, Res} | 211.518/48 | 0.968 | 0.950 | 0.042 | 59.8 | 226.1 | Reverse explanatory |

*Note. Models 0, A, B, and C are saturated (effectively df = 0 once all permitted core paths are estimated; χ² ≈ 0). Their CFI, TLI, and RMSEA values are therefore not informative and are reported as “—”. AIC and BIC do allow comparison: Model 0 estimates more paths and therefore carries a larger penalty than Models A/C, but no model can be statistically declared superior on the basis of fit alone. Model D (parallel mediation) and Model E (reverse explanatory) are not saturated and can be evaluated by standard fit criteria; Model D shows substantially worse fit than the serial models, while Model E shows acceptable fit.*

**Supplementary Table S5. Key indirect effects and path estimates in the hypothesized and alternative models.**

| **Model** | **Effect / pathway** | **Estimate** | **Boot SE** | **CI lower** | **CI upper** | **p** | **Significance** |
| --- | --- | --- | --- | --- | --- | --- | --- |
| **Model 0** | Total effect | 0.1810 | 0.0298 | 0.1253 | 0.2436 | <0.001 | **Significant** |
|  | Direct effect | 0.0120 | 0.0225 | -0.0287 | 0.0592 | 0.594 | NS |
|  | Total indirect effect | 0.1690 | 0.0176 | 0.1348 | 0.2042 | <0.001 | **Significant** |
|  | ACEs → Rumination → Burnout | 0.0335 | 0.0077 | 0.0196 | 0.0497 | <0.001 | **Significant** |
|  | ACEs → Self-control → Burnout | 0.0163 | 0.0069 | 0.0021 | 0.0293 | 0.018 | **Significant** |
|  | ACEs → Resilience → Burnout | 0.0363 | 0.0158 | 0.0050 | 0.0668 | 0.022 | **Significant** |
|  | ACEs → Rumination → Self-control → Burnout | 0.0300 | 0.0067 | 0.0180 | 0.0441 | <0.001 | **Significant** |
|  | ACEs → Rumination → Resilience → Burnout | 0.0275 | 0.0068 | 0.0155 | 0.0421 | <0.001 | **Significant** |
|  | ACEs → Self-control → Resilience → Burnout | 0.0090 | 0.0039 | 0.0011 | 0.0165 | 0.021 | **Significant** |
|  | ACEs → Rumination → Self-control → Resilience → Burnout | 0.0165 | 0.0037 | 0.0099 | 0.0244 | <0.001 | **Significant** |
| **Model A** | Total indirect effect | 0.1657 | 0.0211 | 0.1234 | 0.2053 | <0.001 | **Significant** |
|  | ACEs → Resilience → Burnout | 0.1148 | 0.0174 | 0.0792 | 0.1475 | <0.001 | **Significant** |
|  | ACEs → Rumination → Burnout | 0.0297 | 0.0103 | 0.0101 | 0.0505 | 0.004 | **Significant** |
|  | ACEs → Resilience → Rumination → Burnout | 0.0212 | 0.0037 | 0.0141 | 0.0287 | <0.001 | **Significant** |
| **Model B** | Total indirect effect | 0.1328 | 0.0190 | 0.0973 | 0.1715 | <0.001 | **Significant** |
|  | ACEs → Self-control → Burnout | 0.0718 | 0.0103 | 0.0531 | 0.0934 | <0.001 | **Significant** |
|  | ACEs → Rumination → Burnout | 0.0432 | 0.0121 | 0.0210 | 0.0680 | <0.001 | **Significant** |
|  | ACEs → Self-control → Rumination → Burnout | 0.0177 | 0.0030 | 0.0124 | 0.0240 | <0.001 | **Significant** |
| **Model C** | Total indirect effect | 0.1657 | 0.0211 | 0.1234 | 0.2053 | <0.001 | **Significant** |
|  | ACEs → Rumination → Burnout | 0.0509 | 0.0106 | 0.0313 | 0.0734 | <0.001 | **Significant** |
|  | ACEs → Resilience → Burnout | 0.0582 | 0.0205 | 0.0185 | 0.0980 | 0.005 | **Significant** |
|  | ACEs → Rumination → Resilience → Burnout | 0.0566 | 0.0127 | 0.0336 | 0.0829 | <0.001 | **Significant** |
| **Model D** | Total indirect effect | 0.1690 | 0.0176 | 0.1349 | 0.2042 | <0.001 | **Significant** |
|  | ACEs → Rumination → Burnout | 0.0335 | 0.0077 | 0.0196 | 0.0498 | <0.001 | **Significant** |
|  | ACEs → Self-control → Burnout | 0.0463 | 0.0071 | 0.0337 | 0.0612 | <0.001 | **Significant** |
|  | ACEs → Resilience → Burnout | 0.0893 | 0.0138 | 0.0612 | 0.1150 | <0.001 | **Significant** |
| **Model E** | Burnout → Rumination | 0.4759 | 0.0199 | 0.4368 | 0.5150 | <0.001 | **Significant** |
|  | Burnout → Self-control | -0.5980 | 0.0184 | -0.6341 | -0.5619 | <0.001 | **Significant** |
|  | Burnout → Resilience | -0.6572 | 0.0171 | -0.6908 | -0.6236 | <0.001 | **Significant** |

*Note. All estimates are standardized path coefficients. Boot SE and 95% CI are based on 5,000 nonparametric bootstrap resamples. Significance is determined by whether the 95% CI excludes zero. For Model E, estimates are direct standardized regression coefficients (Burnout → each psychological variable) with normal-theory 95% CIs and Wald p values. Note that the total effect of ACEs on burnout is identical across Models 0/A/B/C/D (β ≈ 0.181), as expected mathematically when the same X, Y, and covariates are used; these models differ only in how the total effect is decomposed into specific pathways.*
